# Review of Echocardiographic Findings in Patients with Obstructive Sleep Apnea

**DOI:** 10.1155/2018/1206217

**Published:** 2018-11-18

**Authors:** Radu Sascău, Ioana Mădălina Zota, Cristian Stătescu, Daniela Boișteanu, Mihai Roca, Alexandra Maștaleru, Maria Magdalena Leon Constantin, Teodor Flaviu Vasilcu, Radu Sebastian Gavril, Florin Mitu

**Affiliations:** ^1^University of Medicine and Pharmacy “Grigore T. Popa”, Iași, Romania; ^2^Institute of Cardiovascular Disease Prof. Dr. George I.M. Georgescu, Iași, Romania; ^3^III^rd^ Pneumology Clinic, Iași, Romania; ^4^Cardiovascular Rehabilitation Clinic, Iași, Romania

## Abstract

Obstructive sleep apnea (OSA) causes recurrent apneas due to upper respiratory tract collapse, leading to sympathetic nervous system hyperactivation and increased cardiovascular risk. Moderate and severe forms of obstructive sleep apnea are associated with increased atrial volumes and affect left ventricular diastolic and then systolic function. Right ventricular ejection fraction can be accurately assessed via three-dimensional echocardiography, while bidimensional imaging can only provide a set of surrogate parameters to characterize systolic function (tricuspid annulus plane systolic excursion, right ventricular fractional area change, and lateral S'). Tissue Doppler imaging is a more sensitive tool in detecting functional ventricular impairment, but its use is limited by angle dependence and the unwanted influence of tethering forces. Two-dimensional speckle tracking echocardiography is considered more suitable for the assessment of ventricular function, as it is able to distinguish between active and passive wall motion. Abnormal strain values, a marker of subclinical myocardial dysfunction, can be detected even in patients with normal ejection fraction and chamber volumes. The left ventricular longitudinal strain is more affected by the presence of obstructive sleep apnea than circumferential strain values. Although the observed OSA-induced changes are subtle, the benefit of a detailed echocardiographic screening for subclinical heart failure in OSA patients on therapy adherence and outcome should be addressed by further studies.

## 1. Introduction

The hallmark of obstructive sleep apnea (OSA) is repetitive collapse of the upper respiratory airways, causing recurrent apneas or hypopneas, intermittent oxygen desaturations, and nocturnal microawakenings. An increase in disease prevalence has been recently detected in the general population, partly attributable to current pandemic obesity. It is estimated that 4% of middle-aged males and 2% of females suffer from OSA [1], a condition that induces refractory hypertension, oxidative stress, endothelial dysfunction, and increased sympathetic tone, leading to increased cardiovascular risk [2]. Obesity, diabetes, and metabolic syndrome are frequently associated with OSA [2, 3]. An overnight sleep study (polysomnography) is the standard diagnostic test for OSA, as it detects the number of apneic and hypopneic episodes, and it allows the calculation of the apnea-hypopnea index (AHI). According to AHI, OSA severity is classified into mild, moderate, and severe forms (AHI 5–14, 15–30, and >30 events/hour, respectively) [4]. Several studies reported a higher incidence of cardiac structural or functional alterations in patients with OSA, which we reviewed in the following sections (continuous variables are expressed as mean values ± SD; a *p* value <0.05 was considered statistically significant).

Continuous positive airway pressure (CPAP) therapy is still considered the gold standard treatment for moderate-to-severe OSA, but its results are limited by inadequate patient adherence to and acceptance of device therapy. A recent report has shown that only 57% of patients diagnosed with moderate-to-severe OSA initiated the recommended CPAP therapy and that only half of them continued to use the device after 1 year [4]. Optimal CPAP therapy requires a minimum of 4 hours of nightly use. Furthermore, up to 83% of patients following CPAP therapy do not reach the recommended threshold of 4 hours of nightly use [5]. Detailed echocardiographic screening for subclinical heart failure could improve clinical outcome in OSA patients by raising their awareness concerning the consequences of sleep-disordered breathing. This could complete the current educational, technological, and psychosocial techniques aimed to improve patient adherence to both device therapy and lifestyle changes [6].

### 1.1. Left Chamber Dimensions

Left atrial enlargement is reported in 18% of newly diagnosed OSA patients [7], being more common among subjects with moderate-to-severe OSA (52.1%) than in patients with the apnea-hypopnea index (AHI) <15 (31%; *p* < 0.001) [8]. The left atrial diameter is higher in patients with severe OSA than in subjects with mild sleep apnea (36.1 ± 5.7 mm versus 32.8 ± 2.3 mm; *p* < 0.01) [9]. Several studies showed that both indexed left atrial volume (LAVI) [10–14] and left atrial area (LAA) [8] increase with OSA severity (Table 1).

OSA is also associated with left ventricular hypertrophy, even in the absence of hypertension, obesity, and diabetes [9, 11] (Table 2). Left ventricular posterior wall (LVPW) thickness, interventricular septum (IVS) thickness, and left ventricular mass (LVM) are reportedly higher in patients with severe OSA than in patients with moderate and mild OSA [9, 16]. Zhou et al. also found a significant difference regarding IVS thickness in subjects with severe and moderate OSA versus controls (*p*=0.001, *p*=0.002, respectively) [15]. The LVM index is significantly higher in subjects with severe OSA than in controls [7, 9], but no statistically significant changes in LV end-systolic and end-diastolic diameters were reported [8, 9]. Left ventricular hypertrophy appears to be correlated to mean nocturnal oxygen saturation. The latter was an independent predictor of left ventricular mass and wall thickness (for every 1% decrease in saturation, the authors reported a mass gain of 4.38 g and an increase in wall thickness by 0.14 cm) [18].

### 1.2. Left Ventricular Systolic Function

Literature reports concerning LV ejection fraction (EF) and LV fractional shortening in OSA patients are controversial. Some publications show normal LV-EF (59 ± 10%) among patients with OSA [9, 16], and several studies reported no significant differences between OSA severity and left ventricular EF or fractional shortening [9–11]. However, a study of 411 men, average age of 71 years old, showed that LV-EF is slightly lower in patients with moderate-to-severe OSA (61.0 ± 8.9%) than in subjects with AHI <15 (62.7 ± 6.3%; *p*=0.028) [8], and another recent report found that OSA severity is significantly correlated with a reduction in LV-EF (*p*=0.005) [19] (Table 3). These results are confirmed by another study of 119 OSA patients monitored over 18 years, showing that, for every tenfold increase in AHI, the left ventricular ejection fraction showed a 1.3% independent decrease [18].

### 1.3. Left Ventricular Diastolic Dysfunction

Previous experimental reports regarding artificially induced OSA in a canine model [20, 21] have shown that each hypoxic episode affects LV diastolic function by increasing left ventricular afterload and that LV systolic dysfunction develops after only 3 months. In humans, OSA-induced systolic and diastolic dysfunction seems to follow a similar pattern, although it typically evolves in a much longer period of time, as it begins by affecting diastolic function, leading to systolic dysfunction only after extended exposure (>10 years) [22, 23]. Nocturnal minimum oxygen saturation <70% was an independent predictor of diastolic dysfunction (OR = 4.34; *p*=0.02) [23]. A recent study reported that 44% of OSA patients with normal biventricular systolic function presented different degrees of diastolic dysfunction [2]. In another report, diastolic dysfunction prevalence was 56.8% among patients with mild OSA, it reached 69.7% in the moderate-to-severe OSA group (*p*=0.002) [16], and Baguet et al. found that 22.7% of subjects with newly diagnosed OSA had mitral flow pattern suggesting impaired LV relaxation [7]. The differences regarding diastolic dysfunction prevalence among subjects with OSA are explained not only by the echocardiographic parameters used in defining it but also by other patient characteristics such as associated comorbidities (obesity and diabetes) and also significant differences regarding OSA severity (the group analyzed by Korcarz et al. included patients with an average AHI 39.8, while the desaturation index in Wachter's moderate-to-severe OSA subgroup was only 20 events/hour).

An increased AHI was associated with decreased mitral E wave [9] and increased mitral A wave velocities [9, 17] (Table 4). Although Varghese et al. did not find a significant difference regarding mitral E/A ratio in patients with severe OSA (1.1 ± 0.2) versus controls (1.2 ± 0.2; *p*=0.09) [10], several other studies reported that mitral E/A ratio decreases with OSA severity [9, 13, 17]. Surprisingly, Altekin et al. found significantly lower E/A ratio only in patients with mild OSA (*p*=0.0009) but not in subjects with moderate or severe OSA [11].

E wave deceleration time (E-DecT) is higher in patients with OSA than in control subjects, but no significant differences regarding E-DecT were found among patients with different degrees of OSA severity [14]. The same trend was reported regarding LV isovolumic relaxation time (IVRT) [14], and it seems that both E-DecT and IVRT have a positive correlation with AHI [9]. Other studies reported significant differences regarding E-Dec and IVRT among OSA subjects (Table 4).

Left ventricular Tei index (LV-MPI), illustrating both systolic and diastolic functions, is higher in subjects with severe sleep apnea (0.64 ± 0.14) compared to those with mild OSA (0.50 ± 0.09; *p* < 0.01) [9] or to controls [14], being a valuable parameter in the early diagnosis of LV dysfunction [9, 24]. Two studies reported a positive correlation between LV-MPI and AHI (*p* < 0.001; *r*=0.825) [9] and (*p*=0.02; *r*=0.27) [12], but other authors found no significant difference regarding LV-MPI between patients with severe OSA and controls (*p*=0.12) [10] (Table 5).

As illustrated above, conventional Doppler echocardiography yields conflicting results regarding OSA impact on left ventricular diastolic function. While several Doppler parameters are not applicable in patients with atrial fibrillation, others are influenced by patient heart rate and blood pressure values or exhibit age dependency. The need of a more comprehensive echocardiographic analysis (that can overcome conventional Doppler limitations) has been recognized by current international echocardiography guidelines, which have included TDI in the diagnostic algorithms of LV diastolic dysfunction [26].

### 1.4. Tissue Doppler Imaging (TDI)

TDI is able to detect subtle changes in systolo-diastolic ventricular function, even in patients with normal EF. The technique is limited by its angle dependence, by the need to acquire high frame rate imaging, and by the influence of myocardial translation and tethering forces. LV-S' wave velocity is an indirect marker of ventricular systolic function. Two studies found no significant difference regarding LV-S' wave velocity among patients with OSA and controls [11, 15], consistent with the controversial reports regarding LV-EF in OSA.

Wachter et al. found that only tissue Doppler-derived parameters (and not mitral flow pattern) are significantly different in the presence of sleep apnea, reporting that lateral e` was significantly lower in subjects with AHI ≥ 15 (7.4 ± 2.1) than in controls (8.3 ± 2.6) [16]. Varghese et al. also showed that subjects with very severe obstructive sleep apnea (AHI >40) present lower e` velocities than controls [10], with similar results being reported by several other authors (Table 6).

E/e` mean ratio, an estimate of LV end-diastolic pressures, is higher in subjects with moderate-to-severe OSA than in controls [10, 11, 16, 25, 27] and is positively correlated to AHI (*r*=0.202; *p*=0.014) [17]. Since E/E` is known to be linked to myocardial fibrosis, Altekin et al. suggest that fibrosis, along with increased LV filling pressures, promotes subclinical LV systolic dysfunction in OSA subjects [11]. Average A' wave velocity and also LA volumes (precontraction and maximum and minimum volumes measured in 3D echocardiography) are higher in patients with severe OSA, as opposed to E'/A' ratio, which decreases with OSA severity [25].

### 1.5. OSA Impact on Right Chambers

Right ventricular dysfunction is a common finding in patients with OSA, and Sanner et al. showed that right ventricular failure is more frequent in patients with OSA even in the absence of any other respiratory conditions [28]. Recent reports showed that the right atrial volume index (RAVI) is higher in subjects with severe OSA than in patients with mild OSA or controls [14].

The Framingham study did not reveal any difference regarding right ventricular (RV) volumes, end-diastolic dimensions (minor axis and major axis in apical view), and systolic function (assessed via right ventricular fractional area change) between subjects with sleep-disordered breathing and controls [29]. Due to the complex anatomical shape of the RV, its end-systolic and end-diastolic volumes cannot be appropriately calculated using 2D echocardiography. RV fractional area change and MPI are surrogate markers that estimate RV global function, while other parameters such as TAPSE and S' have the main disadvantage of offering only a partial representation of RV systolic function [30].

Adequate right ventricular volume estimations are not possible without the 3D technique, and two such studies showed that patients with moderate-to-severe OSA have higher indexed RV end-diastolic and end-systolic volumes compared to controls (*p* < 0.05) [27, 31]. Although some reports found that RV-EF is similar in patients with and without OSA [32], Oliveira et al. have reported a minor but statistically significant difference in RV-EF (Table 7) [27]. A strong association between AHI, RV diameter (*r*=0.482; *p*=0.0009) [34], and RV-EF (*r*=−0.362; *p*=0.02) [31] was also reported. RV wall thickness is higher in subjects with more severe forms of OSA (0.78 ± 0.02 versus 0.68 ± 0.02; *p*=0.005) [29] and is apparently correlated with AHI (*r*=0.356; *p*=0.026) [31].

Although a recent report did not find a significant decrease in the right ventricular Tei index (RV-MPI) in patients with severe sleep apnea [16], a previous study noted a significant difference in RV-MPI between controls and patients with moderate and severe OSA [12]. The same study also found a positive correlation between RV-MPI and AHI (*r*=0.40; *p*=0.002) [12]. These findings are supported by two other reports, showing that RV-MPI is significantly higher in OSA patients than in controls (but not between patients with mild-to-moderate versus severe OSA) [14, 33]. It appears that an increase in AHI has a greater effect on right ventricular global function than on the left ventricular one [14]. Due to ventricular interdependence, the authors speculate that OSA-induced RV dysfunction may contribute to the development of subsequent LV dysfunction [14].

2D echocardiographic parameters that describe global right ventricular function include tricuspid annular plane systolic excursion (TAPSE), myocardial performance index, and right ventricular fractional area change (RV-FAC) [14]. However, tissue Doppler is more sensitive than 2D echocardiography in detecting subclinical RV dysfunction [14]. Although RV E/A ratio does not significantly differ in subjects with moderate-to-severe OSA living at high altitudes compared to healthy controls, the RV E/E` ratio was significantly higher in the OSA group [31]. Altekin et al. did not find significant differences regarding RV A wave velocity or PAP, but he found that E wave deceleration time, A' velocity, and E/E' ratio were higher in subjects with severe OSA compared to controls and subjects with mild OSA [14]. TAPSE, E' wave velocity, and RV E/A ratio were lower in patients with moderate-to-severe OSA than in the control and mild OSA groups [14].

Recent results concerning the impact of OSA on RV S' are controversial, with Dobrowolski et al. stating that it does not significantly differ between patients with and without OSA [35]. Zakhama et al. and Shivalkar et al. observed different results [33], reporting a significant correlation between AHI and RV S` [34]. Although two recent papers did not find any significant differences regarding TAPSE between patients with and without OSA [35, 36], another study reported a correlation between TAPSE and AHI (*r*=−0.285, *p*=0.079) [31].

### 1.6. Pulmonary Hypertension

The prevalence of pulmonary hypertension among patients with OSA ranges between 12% and 70%, depending on OSA severity, PAP assessment method, time of measurement, and other possible confounding factors. Pulmonary hypertension is commonly evaluated via the modified Bernoulli equation to estimate PASP (pulmonary artery systolic pressure) [36], but other formulas have been proposed for calculating mean pulmonary artery pressure (mean PAP) in patients without tricuspid insufficiency [37].

Although Altekin et al. [14] found no significant differences between PASP or mean PAP among patients with different degrees of OSA severity and controls, several other studies showed that patients with moderate and severe OSA present higher PASP than healthy controls [15, 34] (Table 8). They also reported that PASP is significantly higher in patients with moderate-to-severe OSA living at high altitudes than in controls (*p*=0.002) [31]. Pulmonary acceleration time is significantly lower in subjects with moderate-to-severe OSA living at high altitudes versus controls (*p*=0.001) and is directly correlated to AHI (*r*=−0.282; *p*=0.077) [31].

Pulmonary artery (PA) stiffness can be calculated as the ratio between pulmonary artery maximal frequency shift and pulmonary acceleration time (obtained from pulsed-wave Doppler trace of pulmonary artery flow). PA stiffness appears to be correlated both with AHI and mean oxygen saturation [36]. A recent report showed that subjects with OSA present increased PA stiffness even in the absence of pulmonary hypertension [36]. The authors recommend PA stiffness as a more reliable parameter than pulmonary artery pressure in patients with OSA [36].

### 1.7. Other Parameters

Epicardial fat thickness, an indirect marker of visceral adiposity, is significantly higher in patients with moderate or severe OSA (AHI>15). Furthermore, 24 weeks of CPAP treatment induces a significant regression of epicardial fat thickness, even in the absence of any significant changes in BMI or waist circumference [38]. These results are supported by a more recent study that included patients with heart failure (LV-EF <45%) and showed that epicardial adipose thickness was significantly greater in subjects with sleep-disordered breathing than in patients without sleep apnea (10.7 ± 2.8 vs. 8.13 ± 1.8; *p*=0.001) [39].

A previous study showed that OSA is more frequent among patients with Marfan syndrome and found that aortic root diameter is 0.8 cm higher in OSA patients (*p* < 0.0001), suggesting that OSA might contribute to aortic root enlargement in such patients [40].

### 1.8. Speckle Tracking Echocardiography

Strain imaging allows the distinction between active and passive wall motion (tethering). As opposed to tissue Doppler imaging, two-dimensional speckle tracking echocardiography is not angle dependent, providing a more accurate description of global and segmental systolic function. Patients with severe OSA present significantly decreased global longitudinal strain values compared to the other groups [11]. The same report showed a significant difference regarding basal, mid, and apical strain values between subjects with severe OSA and all other groups [11]. Another report showed that global left ventricular longitudinal strain is reduced in patients with very severe OSA [10]. Apical and middle LV segments showed more pronounced longitudinal strain anomalies (Table 9), but circumferential strain did not significantly differ between subjects with very severe OSA and controls [10]. Another recent report found an epicardial-to-endocardial strain gradient at each myocardial level [15]. Only longitudinal (and not circumferential) strain was directly correlated with AHI [15]. Patients with severe OSA presented decreased three-layer longitudinal LV strain, despite having a normal ejection fraction [15].

Subjects with diastolic dysfunction often have abnormal GLS even with normal LV-EF and LV volumes [41]. In such cases, longitudinal myocardial fibers, mainly located in the subendocardium, are very vulnerable to fibrosis, resulting in decreased longitudinal shortening and a compensatory elevation of circumferential shortening and torsion [42]. LV torsion is the twist of the ventricle around its long axis, given by the antagonistic rotation of the basal and apical segments [17], and increased LV torsion is an early indicator of left ventricular dysfunction in patients with normal LV-EF. Reports show contradictory results regarding LV torsion in OSA patients: while Vural et al. found that LV torsion is decreased in subjects with AHI>30 and in controls compared to patients with mild or moderate sleep apnea [17], Vitarelli et al. reported a significant increase in LV torsion in severe sleep apnea versus controls [24].

A recent study showed that left atrial strain rate values are significantly higher in subjects with severe OSA and found a positive correlation between AHI and LA contractile strain, which can be partially improved after 12 weeks of CPAP therapy [17]. While standard tissue Doppler techniques are limited by tethering forces and myocardial translational motion, 2D speckle tracking imaging (2D-STE) is not influenced by Doppler beam angling or load dependency and can provide a more accurate assessment of RV function. RV radial function analysis is subjected to significant errors due to the anterior position of the RV in parasternal views and frequent artifacts, making the RV longitudinal strain and strain rate values to be preferred [14]. Furthermore, as the interventricular septum motion is under the greater influence of the left ventricle, strain and strain rate assessments of the RV should only include the RV free wall as seen in the apical view [43, 44].

The effect of OSA on RV function is controversial. A report by Hammerstingl et al. showed that RV global, apical, and basal longitudinal strains are correlated to the severity of OSA [19], but after multivariate regression analysis, only apical RV longitudinal strain parameters were independently associated with severe sleep apnea [19]. Their study suggests that apical RV longitudinal strain is a sensitive parameter for the diagnosis of subclinical RV dysfunction [16]. However, another report did not show any significant difference concerning segmental RV strain and strain rate between subjects with moderate-to-severe OSA and controls [31]. Patients with moderate-to-severe OSA have lower RV strain and RV systolic strain values than controls or subjects with AHI<15 [14]. The RV early diastolic strain rate decreases with disease severity but the RV late diastolic strain rate increases with AHI [14]. It appears that the 2D speckle tracking parameters correlate better with AHI than any other echocardiographic parameters and should be used in detecting early, subclinical RV dysfunction [14]. The same result is supported by Buonauro et al. [32] who have shown that subclinical RV dysfunction (in patients with normal RV-EF and TAPSE values) can be determined via speckle tracking echocardiography [32].

## 2. Conclusions

There is sufficient evidence to prove that moderate and severe forms of OSA are associated with decreased ventricular function and increased atrial volume, explaining the high incidence of chronic heart failure and also atrial fibrillation in these patients. It seems that OSA begins by affecting diastolic function, leading in time to systolic dysfunction. 2D-STE is not influenced by Doppler beam angling or load dependency. Abnormal strain values, a marker of subclinical systolo-diastolic dysfunction, can be detected even in patients with normal EF and chamber volumes. However, the role of 2D-STE in OSA patients (especially regarding RV function assessment) should be addressed by further studies, as current data yield conflicting results.

## Figures and Tables

**Table 1:** Left atrial volume and area in relationship with OSA severity.

Echocardiographic variables		AHI <5 events·h^−1^	Mild OSA	Moderate OSA	Severe OSA	*p*
LAVI (ml/m^2^)	Altekin et al. [11, 14]	21.6 ± 4.7	22.2 ± 4.8	27.44 ± 6.9^†^	32.3 ± 5.1^†,‡^	<0.03
	Varghese et al. [10]	28.3 ± 4.1			30.6 ± 3.5	0.02
	Romero-Corral et al. [12]	26.8 ± 11	32.5 ± 15^†^	30.4 ± 11^†^		<0.05
	Imai et al. [13]		20.3 ± 4.9		23.3 ± 5.2	<0.0001
LAA (cm^2^)	Hjälm et al. [8]	21.6 ± 4.5		23.7 ± 5.5		<0.001

LAVI: left atrial volume index; LAA: left atrial area; AHI: apnea-hypopnea index; OSA: obstructive sleep apnea; AHI: apnea-hypopnea index; OSA: obstructive sleep apnea. ^†^Significantly different from AHI <5 events·h^−1^; ^‡^significantly different from mild OSA.

**Table 2:** Left ventricular bidimensional parameters in relationship with OSA severity.

Echocardiographic variables		AHI <5 events·h^−1^	Mild OSA	Moderate OSA	Severe OSA	*p*
IVSD (cm)	Altekin et al. [14]	0.89 ± 0.09	1.03 ± 0.1	1.03 ± 0.13^†^	1.13 ± 0.13^†^	<0.05
	Zhou et al. [15]	0.92 ± 0.11	0.97 ± 0.17	1.12 ± 0.16^†^	1.14 ± 0.19^†^	<0.005
	Dursunoglu et al. [9]		0.99 ± 0.09	1.09 ± 0.13^‡^	1.12 ± 0.11^‡^	≤0.01
	Wachter et al. [16]	1.17 ± 0.16	1.22 ± 0.19^†^	1.24 ± 0.18^†^		<0.05
	Holtstrand et al. [8]	1.04 ± 0.14		1.07 ± 0.14		0.028
	Varghese et al. [10]	1.12 ± 0.12			1.18 ± 0.13	0.05
	Vural et al. [17]	0.95 ± 0.11	0.95 ± 0.11	1.00 ± 0.12	1.01 ± 0.11^†^	<0.05
PWD (cm)	Altekin et al. [14]	0.88 ± 0.07	1.03 ± 0.09^†^	1.04 ± 0.12^†^	1.11 ± 0.13^†^	<0.05
	Dursunoglu et al. [9]		0.98 ± 0.08	1.08 ± 0.09^‡^	1.14 ± 0.09^‡^	≤0.01
	Wachter et al. [16]	1.08 ± 0.14	1.12 ± 0.14^†^	1.13 ± 0.14^†^		<0.05
LVMI (g/m^2^)	Varghese et al. [10]	1.07 ± 0.11			1.14 ± 0.13	0.05
	Altekin et al. [11]	86.5 ± 18.7	93.2 ± 16.6	94.5 ± 22.9	103.5 ± 22.9^†^	<0.05
	Varghese et al. [10]	92.4 ± 9.8			98.5 ± 13.5	0.04
	Dursunoglu et al. [9]		100.5 ± 42.3	126.5 ± 41.2^‡^	144.7 ± 39.8^‡^	≤0.002
	Wachter et al. [16]	113 ± 26	119 ± 26	125 ± 29		<0.001
	Imai et al. [13]		107.2 ± 18.5	117.4 ± 19.9		<0.0001
RWT (cm)	Imai et al. [13]		0.4 ± 0.05	0.4 ± 0.04		<0.0001

IVSD: interventricular septum thickness; PWD: posterior wall thickness; LVMI: left ventricular mass index; RWT: relative wall thickness; AHI: apnea-hypopnea index; OSA: obstructive sleep apnea.^†^Significantly different from AHI <5 events·h^−1^; ^‡^significantly different from mild OSA.

**Table 3:** Left ventricular ejection fraction and OSA severity.

Echocardiographic variables		AHI <5 events·h^−1^	Mild OSA	Moderate OSA	Severe OSA	*p*
LV-EF (%)	Hammerstingl et al. [19]		65.0 ± 6.9	59.5 ± 6.9	57.5 ± 5.6	<0.0001
	Holstrand et al. [8]	62.7 ± 6.3	61.0 ± 8.9			0.028

LV-EF: left ventricular ejection fraction; AHI: apnea-hypopnea index; OSA: obstructive sleep apnea.

**Table 4:** Left ventricular diastolic function in relationship with OSA severity.

Echocardiographic variables		AHI <5 events·h^−1^	Mild OSA	Moderate OSA	Severe OSA	*p*
E velocity (cm/s)	Dursunoglu et al. [9]		92 ± 12	77 ± 24	61 ± 13	0.01
A velocity (cm/s)	Dursunoglu et al. [9]		67 ± 25	105 ± 90	87 ± 14	0.01
	Vural et al. [17]	75.1 ± 20.9	78.3 ± 21.5	76.9 ± 13.8	86.2 ± 17.8^†^	<0.05
E/A ratio	Dursunoglu et al. [9]		1.37 ± 0.02^†^	0.73 ± 0.01^‡^	0.70 ± 0.01^‡^	0.01
	Imai et al. [13]		1.38 ± 0.45		1.03 ± 0.39	<0.0001
	Vural et al. [17]	1.0 ± 0.3	1.0 ± 0.3	1.0 ± 0.3	0.8 ± 0.2^†^	<0.05
DecT (ms)	Altekin et al. [11]	1.19 ± 0.24	0.96 ± 0.16^†^	1.01 ± 0.3	1.11 ± 0.28	0.009
	Altekin et al. [11]	163 ± 26.2	227.4 ± 31.1^†^	216.4 ± 60.4^†^	199.4 ± 39.5^†^	<0.001
	Dursunoglu et al. [9]		170.1 ± 20.9	210.0 ± 47.7	240.1 ± 57.7	0.01
	Imai et al. [13]		187.1 ± 33.7		198.4 ± 36.6	0.003
	Vitarelli et al. [24]	143 ± 14	175 ± 17		229 ± 15^†^	0.004
	Oliveira et al. [25]		189.2 ± 34.5	231.8 ± 45.2	247.6 ± 48.2^‡^	<0.05
IVRT (ms)	Altekin et al. [11]	88.3 ± 12.5	106.3 ± 12.8^†^	108.8 ± 12.9	113.2 ± 10.4^†^	<0.001
	Vitarelli et al. [24]	74 ± 11	103 ± 9		125 ± 10^†^	0.004
	Fung et al. [23]	92.7 ± 16.6			106.4 ± 19.1	0.005
	Dursunoglu et al. [9]		72.0 ± 12.6	100.2 ± 13.7^‡^	125.5 ± 13.1^‡^	0.01

DecT: deceleration time; IVRT: isovolumic relaxation time; AHI: apnea-hypopnea index; OSA: obstructive sleep apnea. ^†^Significantly different from AHI <5 events·h^−1^; ^‡^significantly different from mild OSA.

**Table 5:** Left ventricular myocardial performance index in relationship with OSA severity.

Echocardiographic variables		AHI <5 events·h^−1^	Mild OSA	Moderate OSA	Severe OSA	*p*
LV-MPI	Dursunoglu et al. [9]		0.50 ± 0.09	0.60 ± 0.10 ^‡^	0.64 ± 0.14^‡^	0.01
	Altekin et al. [14]	0.46 ± 0.08	0.48 ± 0.08	0.55 ± 0.06^†^	0.6 ± 0.13^†,‡^	<0.05
	Vitarelli et al. [24]	0.39 ± 0.08	0.43 ± 0.07		0.58 ± 0.09^†^	0.039

LV-MPI: left ventricular myocardial performance index; AHI: apnea-hypopnea index; OSA: obstructive sleep apnea. ^†^Significantly different from AHI <5 events·h^−1^; ^‡^significantly different from mild OSA.

**Table 6:** Left ventricular tissue Doppler parameters in patients with OSA.

Echocardiographic variables		AHI <5 events·h^−1^	Mild OSA	Moderate OSA	Severe OSA	*p*
E' (cm/s)	Imai et al. [13]		10.7 ± 3.0		8.7 ± 1.9	<0.0001
	Varghese et al. [10]	10.6 ± 1.1			9.2 ± 2.1	0.01
	Vural et al. [17]	11.6 ± 1.5	9.6 ± 1.7	6.3 ± 1.1^†,‡^	6.1 ± 1.4^†,‡^	<0.05
	Oliveira et al. [25]		7.7 ± 1.5	7.3 ± 2.0	6.2 ± 1.8^‡,^^*∗*^	<0.05
	Vitarelli et al. [24]	12.1 ± 2.2	11.4 ± 2.1		7.7 ± 2.8^†^	0.016
E/E`	Altekin et al. [11]	6.88 ± 1.7	6.91 ± 1.9	8.56 ± 2.37	10.29 ± 1.48^†,‡,^^*∗*^	<0.001
	Wachter et al. [16]	11.0 ± 3.6	11.7 ± 3.5	12.7 ± 5.3^†^		<0.05
	Imai et al. [13]		7.6 ± 2.2		7.8 ± 2.3	0.04
	Varghese et al. [10]	8.44 ± 1.6			9.69 ± 2.6	0.03
A' (cm/s)	Altekin et al [14]	8.13 ± 2.22	8.62 ± 2.68	11.31 ± 2.87^†,‡^	13.89 ± 2.32^†,‡,^^*∗*^	<0.05
	Vural et al. [17]	6.5 ± 1.2	8.4 ± 2.4^†^	11.9 ± 2.2^†,‡^	12.0 ± 2.8^†,‡^	<0.05
	Vitarelli et al. [24]	5.6 ± 1.6	5.5 ± 1.8		8.8 ± 2.5^†^	0.025
	Oliveira et al. [25]		9.7 ± 1.9	10.4 ± 3.1	11.6 ± 3.6^‡,^^*∗*^	<0.05
	Oliveira et al. [27]	9.4 ± 2.9	10.6 ± 3.0			0.02
	Oliveira et al. [25]		6.1 ± 1.4	6.8 ± 1.7	8.2 ± 1.9^‡,^^*∗*^	<0.05
E'/A'	Oliveira et al. [25]		1.3 ± 0.4	1.1 ± 0.3	0.8 ± 0.3^‡,^^*∗*^	<0.05

AHI: apnea-hypopnea index; OSA: obstructive sleep apnea. ^†^Significantly different from AHI <5 events·h^−1^; ^‡^significantly different from mild OSA; ^*∗*^significantly different from moderate OSA.

**Table 7:** Right atrial and ventricular parameters in relationship with OSA.

Echocardiographic variables		AHI <5 events·h^−1^	Mild OSA	Moderate OSA	Severe OSA	*p*
RAVI (ml/m^2^)	Altekin et al. [14]	15.56 ± 4.94	17.31 ± 6.24	21.89 ± 7.91	28.85 ± 7.97^†,‡^	<0.05
RVESV index (ml/m^2^)	Guvenc et al. [31]	22.15 ± 3.85		26.50 ± 8.11		0.01
	Oliveira et al. [27]	15.4 ± 3.6	18.7 ± 4.3			<0.05
RVEDV index (ml/m^2^)	Guvenc et al. [31]	41.48 ± 6.45		48.15 ± 11.48		0.009
	Oliveira et al. [27]	49.9 ± 6.0	52.2 ± 7.3			0.02
RV-EF (%)	Oliveira et al. [27]	68.4 ± 5.9	64.3 ± 6.8			<0.01
TAPSE (mm)	Altekin et al. [14]	24.76 ± 1.55	22.30 ± 2.39	21.11 ± 1.56^†^	19.42 ± 1.64^†,‡,^^*∗*^	<0.05
	Zakhama et al. [33]			26.1 ± 3	22.7 ± 4	0.012
RV-MPI	Romero-Corral et al. [12]	0.23 ± 0.10	0.26 ± 0.16^†^	0.37 ± 0.11^†^		<0.05
	Altekin et al. [14]	0.43 ± 0.09	0.46 ± 0.09	0.53 ± 0.08^†^	0.56 ± 0.11^†,‡^	<0.05
S`RV (cm/s)	Shivalkar et al. [34]	0.25 ± 0.03	0.29 ± 0.05			0.008
	Zakhama et al. [33]	0.46 ± 0.14	0.55 ± 0.12			0.024
	Shivalkar et al. [34]	13.5 ± 1.8	11.4 ± 2.3			<0.001
RV E/E`	Zakhama et al. [33]	14.5 ± 3	12.2 ± 2			<0.001
	Guvenc et al. [31]	3.83 ± 1.16		5.23 ± 2.58		0.008
	Altekin et al. [14]	4.19 ± 1.22	4.37 ± 1.22	5.77 ± 1.27^†,‡^	7.12 ± 2.29^†,‡^	<0.05

RAVI: right atrial volume index; RVESV: right ventricular end-systolic volume; RVEDV: right ventricular end-diastolic volume; RV-EF: right ventricular ejection fraction; TAPSE: tricuspid annular plane excursion; RV-MPI: right ventricular myocardial performance index; AHI: apnea-hypopnea index; OSA: obstructive sleep apnea. ^†^Significantly different from AHI <5 events·h−1; ^‡^significantly different from mild OSA; ^*∗*^significantly different from moderate OSA; ^#^significantly different in OSA compared to controls.

**Table 8:** Pulmonary hypertension parameters in relationship with OSA severity.

Echocardiographic variables		AHI <5 events·h^−1^	Mild OSA	Moderate OSA	Severe OSA	*p*
PASP (mmHg)	Guvenc et al. [31]	30.94 ± 6.47		38.35 ± 8.6		0.002
	Zhou et al. [15]	16.7 ± 6.2	18.2 ± 6.6	31.2 ± 5.6^†^	32.8 ± 6.7^†^	<0.05
PAT (ms)	Shivalkar et al. [34]	22 ± 8	32 ± 10			0.004
	Guvenc et al. [31]	118.36 ± 16.36		103.13 ± 18.42		0.001

PASP: pulmonary artery systolic pressure; PAT: pulmonary artery acceleration time; AHI: apnea-hypopnea index; OSA: obstructive sleep apnea. ^†^Significantly different from AHI <5 events·h^−1^.

**Table 9:** Left and right ventricular strain parameters in subjects with OSA.

Echocardiographic variables		AHI <5 events·h^−1^	Mild OSA	Moderate OSA	Severe OSA	*p*
LV GLS (%)	Varghese et al. [10]	−19 ± 1.6			−15 ± 1.8	<0.01
	Vural et al. [17]	−22.3 ± 4.0	−20.0 ± 2.3	−17.2 ± 2.0^†^	−15.6 ± 5.6^†,‡^	<0.05
LS basal strain (%)	Vitarelli et al. [24]	−21.9 ± 2.8	−21.2 ± 2.5		−18.4 ± 2.7^†^	0.011
	Altekin et al. [11]	−25.6 ± 2.2	−23.9 ± −3.9	−21.3 ± 2.6^†,‡^	−16.9 ± −2.7^†,‡,^^*∗*^	<0.03
	Varghese et al. [10]	−17 ± 1.7			−15 ± 1.9	0.02
	Altekin et al. [11]	−21.4 ± −1.9	−19.9 ± −2.4	−18.4 ± 2.6^†^	−15.3 ± −2.9^†,‡,^^*∗*^	<0.03
LS midstrain (%)	Varghese et al. [10]	−18 ± 1.8			−15 ± 2.3	<0.01
LS apical strain (%)	Altekin et al. [11]	−23.3 ± −2.1	−21.5 ± −2.4	−19.7 ± 2.6^†^	−16.9 ± −2.9^†,‡,^^*∗*^	<0.03
	Varghese et al. [10]	−20 ± 1.7			−16 ± 2.5	<0.01
	Altekin et al. [11]	−27.4 ± −3.7	−24.6 ± −2.9	−23.5 ± 3.9^†^	−19.2 ± −3.9^†,‡,^^*∗*^	<0.03
LV radial strain (%)	Vural et al. [17]	45.7 ± 6.1	44.4 ± 7.7	39.8 ± 7.8^†^	39.8 ± 8.9^†^	<0.05
LV circumferential strain (%)	Vural et al. [17]	−21.6 ± 3.5	−21.2 ± 1.8	−19.3 ± 2.8^†^	−18.8 ± 2.7^†^	<0.05
LV apical rotation (°)	Vural et al. [17]	8.6 ± 1.0	8.7 ± 1.7	9.1 ± 1.1	7.4 ± 1.3^†,‡,^^*∗*^	<0.05
LV torsion (°)	Vural et al. [17]	15.6 ± 1.5	16.1 ± 1.9	16.5 ± 1.6	14.8 ± 1.6^‡,^^*∗*^	<0.05
2D global RV SI	Hammerstingl et al. [19]		−21.5 ± 6.3	−14.3 ± 5.3	−14.5 ± 8.2	<0.0001
	Buonauro et al. [32]	22.8 ± 3.3	20.9 ± 4.9			<0.05
2D apical RV SI	Hammerstingl et al. [19]		−17.3 ± 8.7	−9.8 ± 6.0	−6.3 ± 5.7	<0.0001
2D basal RV SI	Hammerstingl et al. [19]		−27.4 ± 13.6	−18.2 ± 8.7	−21.6 ± 14.9	0.03
RV strain (%)	Altekin et al. [14]	−34.05 ± −4.29	−31.4 ± −5.37	−22.75 ± −4.89^†,‡^	−20.89 ± −5.59^†,‡^	<0.05
RV systolic strain rate	Altekin et al. [14]	−2.93 ± −0.64	−2.85 ± −0.73	−2.06 ± −0.43^†,‡^	−1.43 ± −0.33^†,‡,∗^	<0.05
RV early diastolic strain rate	Altekin et al. [14]	2.38 ± 0.63	2.32 ± 0.84	1.66 ± 0.55^†^	1 ± 0.54^†,‡^	<0.05
RV late diastolic strain rate	Altekin et al. [14]	2.25 ± 0.33	2.32 ± 0.54	2.79 ± 0.66^†^	3.29 ± 0.54^†,‡^	<0.05

LV GLS: left ventricular global longitudinal strain; LS basal: left ventricular basal longitudinal strain; LS mid: left ventricular longitudinal strain in medium segments; LS apical: left ventricular apical longitudinal strain; LV: left ventricular; 2D global RV SI: bidimensional global right ventricular strain index; 2D apical RV SI: bidimensional apical right ventricular strain index; 2D basal RV SI: bidimensional basal right ventricular strain index; RV: right ventricular; AHI: apnea-hypopnea index; OSA: obstructive sleep apnea. ^†^Significantly different from AHI <5 events·h−1; ^‡^significantly different from mild OSA; ^*∗*^significantly different from moderate OSA.
